# Stewarding waste and antimicrobials: an assessment of chorioamnionitis regimens

**DOI:** 10.1017/ash.2025.10156

**Published:** 2025-10-09

**Authors:** Pamela Bailey, Alexia Foy-Crowder, Joseph Kohn, Benjamin Ereshefsky, Amber Shaye Beville, Sean Stuart, Amy Crockett

**Affiliations:** 1 University of South Carolina School of Medicine (Columbia), Columbia, SC, USA; 2 Department of Internal Medicine, Division of Infectious Diseases, Prisma Health Midlands, Columbia, SC, USA; 3 Department of Pharmacy, Prisma Health Midlands, Columbia, SC, USA; 4 Department of Pharmacy, Vanderbilt University Medical Center, Nashville, TN, USA; 5 Infection Prevention, Prisma Health Midlands, Columbia, SC, USA; 6 Department of Obstetrics and Gynecology, Division of Maternal-Fetal Medicine, Prisma Health Upstate, Greenville, SC, USA; 7 University of South Carolina School of Medicine (Greenville), Greenville, SC, USA

## Abstract

Stewardship is not only about appropriate antibiotic prescribing, but also improving the utilization of resources. While multiple treatment regimens are endorsed for chorioamnionitis, some produce significantly more plastic waste than others. Ampicillin with gentamicin ± clindamycin, and vancomycin with gentamicin both create more plastic waste compared to regimens like cefoxitin, piperacillin/tazobactam, or ertapenem.

## Introduction

Antimicrobial resistance (AMR) is an urgent international public health threat, with greater than 2.8 million antimicrobial-resistant infections occurring within the US each year resulting in 35,000 deaths.^1^ Climate change is also an urgent public health threat, with the healthcare sector contributing ∼ 5% of greenhouse gas emissions worldwide.^2,3^ Most emissions (70–80%) in the healthcare sector come from indirect emissions—the supply chain or procurement of pharmaceuticals, medical products, and devices.^3^ Both public health crises demand urgent actions, both direct and indirect, by healthcare providers and healthcare systems in utilization of resources. Antimicrobial-associated greenhouse gas (GHG) emissions may be a powerful tool to help influence prescribing practices and impact antimicrobial and diagnostic stewardship strategies.^4,5^

Chorioamnionitis provides a clear example of this dual goal in stewardship. It is the most common infection-related diagnosis in labor and delivery, affecting 1–6% of term gestations, with significant maternal and neonatal morbidity, requiring urgent administration of intravenous (IV) antimicrobials for presumed polymicrobial infections.^6^

The American College of Obstetricians and Gynecologists (ACOG) endorses multiple potential regimens for chorioamnionitis, primarily guided by penicillin allergies and their severity and accommodating to local microbiology patterns (Table 1).^7^ In 2022–2023, for both stewardship and antimicrobial shortage reasons, both Prisma Health and Vanderbilt University adapted their chorioamnionitis regimens away from the primary regimen of ampicillin/gentamicin ± clindamycin to ACOG-endorsed alternatives of cefoxitin and piperacillin/tazobactam, respectively.^8,9^ Qualitative feedback at both institutions indicated that the nursing workflow was simplified with monotherapy, which sustained the changes after the shortages were resolved and also drove additional hypotheses regarding additional benefits with these regimens, specifically single-use plastics in medications and intravenous administration and reducing the carbon footprint of treating this infection.

**Table 1.** Intraamniotic infection treatment regimens and cumulative number of doses

IAI Regimens^*^	Guideline Recommendation	Number of doses in typical 24-hour period	Weight of individual Medication	Total Weights of Medication Administered in 24h period	FY 2024 (n = 1 074)
Primary Regimen	Ampicillin 2 g every 6h and gentamicin 2 mg/kg IV load followed by 1.5 mg/kg every 8h OR 5 mg/kg every 24h	4 doses ampicillin plus either 3 doses or 1 dose of gentamicin = 7 or 5	4 doses ampicillin = 708 g 3 doses of gentamicin† 2 mg/kg then 1.5 mg/kg = 480 g 1 dose of gentamicin† 5 mg/kg = 540 g	1.19 kg or 1.25 kg	1 278 kg or 1343 kg (1.4 or 1.5 ton)
Mild penicillin allergy	Cefazolin 2 g every 8h and gentamicin 2 mg/kg IV load followed by 1.5 mg/kg every 8h OR 5 mg/kg every 24h	3 doses cefazolin plus either 3 doses or 1 dose of gentamicin = 6 or 4	3 doses of cefazolin = 144 g 3 doses of gentamicin† 2 mg/kg then 1.5 mg/kg = 480 g 1 dose of gentamicin† 5 mg/kg = 540 g	0.62 kg or 0.68 kg	666 kg or 730 kg (0.73 or 0.80 ton)
Severe penicillin allergy	Clindamycin 900 mg every 8h and gentamicin 2 mg/kg IV load followed by 1.5 mg/kg every 8h OR 5 mg/kg every 24h	3 doses of clindamycin plus either 3 doses or 1 dose of gentamicin = 6 or 4	3 doses of clindamycin = 204 g 3 doses of gentamicin† 2 mg/kg then 1.5 mg/kg = 480 g 1 dose of gentamicin† 5 mg/kg = 540 g	0.68 kg or 0.74 kg	730 kg or 795 kg (0.80 to 0.88 ton)
	Vancomycin 1 g every 12h and gentamicin 2 mg/kg IV load followed by 1.5 mg/kg every 8h OR 5 mg/kg every 24h	2 doses of vancomycin plus either 3 doses or 1 dose of gentamicin = 5 or 3	2 doses of vancomycin = 698 g 3 doses of gentamicin† 2 mg/kg then 1.5 mg/kg = 480 g 1 dose of gentamicin† 5 mg/kg = 540 g	1.18 kg or 1.24 kg	1 267 kg or 1 332 kg (1.40 ton or 1.47 ton)
Alternative IAI regimens:					
	Ampicillin/sulbactam 3 g every 6 hours	4 doses	4 doses Ampicillin/ sulbactam = 716 g	0.72 kg	773 kg (0.85ton)
	Piperacillin/tazobactam 3.375 g every 6 hours or 4.5 g every 8 hours	4 or 3 doses	Piperacillin/tazobactam 3.375 g for 4 doses = 480 g Piperacillin/tazobactam 4.5 g for 3 doses = 450 g	0.48 kg or 0.45 kg	516 kg or 483 kg (0.57 ton or 0.53 ton)
	Cefotetan 2 g every 12h‡	2 doses			
	Cefoxitin 2 g every 8h	3 doses	Cefoxitin 2 g for 3 doses = 513 g	0.51 kg	548 kg (0.60 ton)
	Ertapenem 1 g every 24h	1 dose	Ertapenem 1 g for 1 dose = 95 g	0.095 kg	102 kg (0.0.11 ton)

* *ACOG Committee Opinion 712. Intrapartum Management of Intraamniotic Infection* *Postcaesarean delivery: One additional dose of the chosen regimen. Add clindamycin 900 mg IV or metronidazole 500 mg for at least one additional dose.* *Post vaginal delivery: No additional doses required; but if given, clindamycin is not indicated.*

† Using the assumption of a weight of 80 kg for a term pregnant person for weight-based calculations.

‡ We do not carry cefotetan and could not make measurements, but it is included as an ACOG-endorsed regimen.

## Methods

We verified standard procedures at our healthcare systems for all ACOG-endorsed chorioamnionitis dosing regimens, both in the pharmacy and with nursing. Most antibiotics require reconstitution at the bedside, with the exceptions of clindamycin, which is premixed and delivered from pharmacy to the bedside in an infusion bag, and weight-based gentamicin, which is prepared in the clean room. We weighed each individual item for each treatment regimen on a medical grade scale. When weighing medications, the diluents for reconstitution were included in weights of medication as there are indirect emissions in getting the diluents to the bedside from the manufacturer, which contribute to total emissions. We subsequently used online calculators to convert the total estimated waste (kg) into CO_2_ emissions.^10,11^ Delivery data from fiscal year 2024 was used to estimate the study population.

## Results

Prisma Health has 8 hospitals with obstetric services in South Carolina with 16,209 births in fiscal year 2024 and Vanderbilt University Hospital in Tennessee reported 5,283 births in the same time frame. We estimated that 5%, approximately 810 patients and 264 patients (*n* = 1,074), would require treatment in the respective health systems based on the clinical experiences at our institutions (unpublished data).

Estimates of medical waste are provided in Table 1. The primary ACOG-endorsed regimen of ampicillin with gentamicin generated either 1.19 kg or 1.25 kg of plastic waste every 24 hours, depending on the weight-based dosing strategy, generating 1.4–1.5 tons of waste per 24h period of antimicrobials per year on average. The regimen of vancomycin and gentamicin, suggested for “severe penicillin allergy,” generates a similarly-large amount of waste. Most of the alternative regimens (ie, cefazolin with gentamicin, clindamycin with gentamicin, ampicillin/sulbactam, piperacillin/tazobactam, cefoxitin) generate approximately 0.5 tons of waste. Ertapenem, with its once-daily administration, comes in at the lowest waste-generation at 95 g per dose, averaging 0.11 tons per day of antimicrobials per year.

The solid waste generated from 1.5 tons of non-biohazardous materials daily generated 547.5 tons of carbon dioxide equivalents (CO_2_e) per year, including hauling to the landfill.^10^ This translates to the ability to power 67 homes for one year, or 1,264,834 miles driven by an average gasoline-powered passenger vehicle.^11^ This would be 34 trips around the equator or 4 trips to the moon. This total would require 42,221 trash bags of waste recycled instead of discarded in a landfill, or require 8,213 tree seedlings growing for 10 years to sequester an equivalent amount of carbon.

## Conclusions

In these two healthcare systems with 21,492 deliveries in one year, we estimate that the administration of a traditional ampicillin and gentamicin regimen generated greater than 1.5 tons of trash per day with the yearly generation of waste totaling 547.5 tons CO_2_e. This is likely an underestimate since we used a baseline of 24 hours of antimicrobial administration and in clinical practice treatment frequently extends beyond this time frame. Additionally, if patients need a new IV line started for antimicrobial administration, there is a significant additional contribution of both biohazardous and plastic waste from the placement of the IV and fresh plastic tubing. Additional considerations include vancomycin or gentamicin levels, requiring additional lab draws for monitoring if used for prolonged periods of time.

Quantifying the impact of greenhouse gas emissions on healthcare decision-making can have other positive effects. As noted in both institutions when implementing these regimen changes to cefoxitin or piperacillin/tazobactam, moving away from ampicillin/gentamicin ± clindamycin as first line therapy requires multidisciplinary input, education, and buy-in; this greenhouse gas emission and plastic waste consideration may help as another argument for antimicrobial stewards looking to achieve change in their institutions.

While antimicrobial stewardship focuses on optimal antimicrobial use for a particular disease state, another consideration is the frequency of administration of antimicrobials for reducing medication errors as well as nursing time. Ampicillin/gentamicin ± clindamycin may require 11 administrations daily with each IV administration requiring an average of 22 minutes of nursing time.^8^ Use of regimens that promote stewardship of nursing time will nearly always also promote stewardship of non-reusable resources and vice versa, compounding their beneficial properties. We must strike a balance, however: ertapenem, with its once daily administration clearly stewards resources well, but is very broad-spectrum and is not necessarily the best antimicrobial stewardship option; alternatively, ampicillin/sulbactam generates less waste than other regimens but may be too narrow with growing AMR patterns in gram-negative infections. Regimens such as cefoxitin, piperacillin/tazobactam, and cefazolin with gentamicin may represent the middle ground between antimicrobial and resource stewardship.

Innovative research methods are necessary to address the contributions of healthcare work to greenhouse gas emissions. Although we are striving for accuracy in this report, there are limitations. The calculations are based on averages, which may not be applicable in other healthcare systems. Additionally, while we have applied municipal waste and autoclaving as is strictly-indicated regarding potential biohazardous waste, it is important to acknowledge that many items may end up in the wrong waste stream depending on where the practitioner places the item to be disposed. Approximately 30% of healthcare waste is plastics, particularly in intensive care and anesthesia settings—complicated by contamination or infectious issues, which then turn them into hazardous waste requiring incineration as the predominant disposal method.^12^ Material waste in single-use plastics contributes a significant proportion of GHG generated from medical products, compounded by potential disposal issues—incineration instead of recycling.^3,13^

Finally, there is the concern that up to 30% of healthcare interventions in the United States are not indicated or wasteful, such as overtreatment or low-value care, with estimated costs of $760–935 billion and projected potential savings of 25% if waste were addressed.^14^ Incorporating greenhouse gas emmissions data may be a powerful tool to help reduce low-value care and make more compelling arguments regarding stewardship strategies.

## Data Availability

Data available on request.
